# Clustering Approach to Quantify Long-Term Spatio-Temporal Interactions in Epileptic Intracranial Electroencephalography

**DOI:** 10.1155/2007/83416

**Published:** 2007-11-13

**Authors:** Anant Hegde, Deniz Erdogmus, Deng S. Shiau, Jose C. Principe, Chris J. Sackellares

**Affiliations:** ^1^Computational NeuroEngineering Laboratory, Department of Electrical & Computer Engineering, University of Florida, Gainesville, FL 32611, USA; ^2^Department of Computer Science and Electrical Engineering CSEE, OGI School of Science & Engineering, Oregon Health & Science University, Portland, Beaverton, OR 97006, USA; ^3^Optima Neuroscience, Inc., Gainesville, FL 32601, USA; ^4^Malcolm Randal VA Medical Center, Gainesville, FLa, FL 32608, USA

## Abstract

Abnormal dynamical coupling between brain structures is believed to be primarily responsible for the generation of epileptic seizures and their propagation. In this study, we attempt to identify the spatio-temporal interactions of an epileptic brain using a previously proposed nonlinear dependency measure. Using a clustering model, we determine the average spatial mappings in an epileptic brain at different stages of a complex partial seizure. Results involving 8 seizures from 2 epileptic patients suggest that there may be a fixed pattern associated with regional spatio-temporal dynamics during the interictal to pre-post-ictal transition.

## 1. INTRODUCTION

There is sufficient evidence to believe that the brain dynamics can be effectively modeled through complex nonlinear interactions. Application of nonlinear dynamical measures [1, 2] such as short-term Lyapunov exponents (STLmax) and correlation dimension on an epileptic brain have revealed that the complexity of the brain dynamics reduces significantly as a seizure is approaching. In other words, the temporal dynamics of the brain progresses from a “high-dimensional” nonconvergent (chaotic) state to a much smaller dimensional “chaotic” state.

Much of the analysis on temporal dynamics focuses on analyzing and characterizing the irregular behavior of the time signal of either intracranial or scalp EEG. However, it is important to realize that the brain is a multidimensional system with a large set of neuronal oscillators that are physically and functionally coupled together. Obviously, neurons communicate with each other through synaptic potentials resulting in microscopic action potential discharges. Abnormal neural population synchrony can also produce mesoscopic transient activity, clinically called sharp waves or spikes. Depending on the pathophysiological states, the nature of the spikes with respect to their frequency of occurrence, amplitude, and shape, can be very distinctive. Particularly in an epileptic brain, it would be natural to expect the distinctions between interictal, preictal, and ictal spikes could possibly be a consequence of the dynamical changes in spatio-temporal communications between various regions of the brain. Therefore, it is essential to unravel the functional connectivity of the neural networks and analyze how the structures change during seizure events.

Even though observations that the macroscopic EEG cannot be distinguished from linearly correlated noise [3], many nonlinear approaches have been able to extract interregional coupling information in a manner that would not have been possible by spectral approaches. Nonlinear dependencies between multiple signals have been studied in the last two decades, with the hope of enhancing the tool set provided by the linear methods. Unfortunately, they have faced some practical implementation problems such as sensitivity to noise, choice of parameters, and the high computational cost. Most of the state-space methods rely on finding the functional dependencies between two-time series based on how their trajectories in the embedded phase space describe each other. Inspired by the similarity–index (SI) technique introduced by Arnhold et al. [4], we earlier proposed a self-organizing map (SOM)-based computationally efficient measure, SOM-SI [5, 6], to measure asymmetric dependencies between time sequences. Conceptually, the SI and the SOM-SI methods rely on the assumption that if there is a functional dependency between two signals, the neighboring points in the state space of one signal correspond to neighborhoods of their counterpart. The SOM-SI method maps the embedded data from signals onto a quantized output space through an SOM [7, 8] specialized on these signals, and utilizes the activation of SOM processing elements (PE) to infer about the influence directions between the signals. This approach reduces the computational complexity drastically by exploiting the accurate quantization properties of the SOM in representing the dynamics of the signal in the phase space. Our previous work [6] showed that the SOM-SI was capable of determining the temporal evolution of dependencies between various cortical sites, at different stages of temporal lobe epileptic seizures.

Epileptic seizures, in particular, are characterized by dynamic states (interictal, ictal, preictal, and postictal) that are known to possess both local and global spatio-temporal groupings. Channels associate and deassociate in time; however, depending on the psycho-physiological state of the brain, certain groups of channels might have a higher likelihood of sharing same channel connectivities, thus forging a long-term association. In epileptic intracranial EEG, identifying such state-dependent clusters may provide us with useful insights on the evolution of brain patterns during seizure states. In this study, we propose a spatio-temporal clustering model to qualitatively analyze the spatio-temporal groupings in multidimensional epileptic structures. Unlike in many other clustering approaches, where dynamical features extracted from the data are used as basis to determine groupings, our proposed clustering approach uses the dependencies among the original data recordings to do the same. Our approach, in short, essentially seeks to analyze the regional grouping of cortical sites at different stages of a seizure, based on their mutual interactions.

On a clinical perspective, this study intends to investigate spatio-temporal relationships across various regions of an epileptic brain to help determine the epileptic focus and the dynamical changes that lead to a seizure. In order to achieve this ultimately goal, it is necessary to develop appropriate signal processing tools that extract features to cluster different regions of the brain based on their functional dependencies. The highlight of this clustering measure is that it uses a similarity or a proximity matrix that is entirely data-dependent to determine regional dependencies. Our idea is two folds: (a) to propose a novel tool to determine clusters and present synthetic simulations and real data to support the validity and robustness of this measure, (b) to apply this measure on real-epileptic data and present a detailed clinical investigation on the outcome. The study was made on 8 complex partial seizures from 2 patients suffering from temporal lobe epilepsy. The conclusions of this paper are based on observations from these 8 seizures only.

The paper is organized as follows. We first present a brief review of SOM-SI in Section 2. Section 3 discusses the spectral-clustering approach and the proposed spatio-temporal cluster model. Data description is provided in Section 4 followed by clinical evaluation of the clustering approach on the epileptic EEG data, in Section 5. Section 6 discusses about potential directions for future study.

## 2. SIMILARITY INDEX (SI) MEASURE

**(A) Original SI measure**

Assume that X and Y are two time series generated by a system, which are embedded into two vector signals in time using delays. N(X∣Y) is defined as the average dependency of X on Y and it can be written as [5],

(1) $$N(X|Y)=\frac{1}{N}\overset{N-1}{\underset{n=0}{\sum }}\frac{R^{n}(X)-R^{n}(X|Y)}{R^{n}(X)},$$

where $R^{n}\left(X\right)$ is the average Euclidean distance between the state-vector of *$X^{n}$* and the remaining state-vectors in X. The Y-conditioned Euclidean distance $R^{n}\left(X|Y\right)$ measures the average Euclidean distance between *$X^{n}$* and the vectors in X whose corresponding time partners are the k-nearest neighbors of *$Y^{n}$*. This measure takes values in [0,1], where 0 implies no coupling and 1 implies perfect synchronization [4]. Average dependence of Y on X, N(Y∣X), is similarly computed. The difficulty with this approach is that at every time instant n, we must search for the k nearest neighbors of the current embedded signal vectors among all N sample vectors; this process requires *$O\left(N^{2}\right)$* operations. This high complexity hinders real-time implementation and analysis. In addition, the measure depends heavily on the free parameters, namely, the number of nearest neighbors and the neighborhood size ε. The neighborhood size **ε** needs to be adjusted every time the dynamic range of the windowed data changes.

### 2.1. SOM-based similarity index (SOM-SI)

The self-organized-map- (SOM-) based SI algorithm [5] is fundamentally aimed at reducing the computational complexity of the SI technique. The central idea is to create a statistically quantized representation of the dynamical system using an SOM [7, 8]. An SOM is a neural-network in which spatial patterns from the input space are mapped onto an ordered output space consisting of a set of processing elements (PE). Thus each PE in the SOM, based on its location on the map, compactly models different features/dynamics of the input.

For best generalization, the map needs to be trained to represent all possible states of the system (or at least with as much variation as possible). As an example, if we were to measure the dependencies between EEG signals recorded from different regions of the brain, it is necessary to create an SOM that represents the dynamics of signals collected from all channels. The SOM can then be used as a prototype to represent any signal recorded from any spatial location on the brain, assuming that the SOM PEs have specialized in the dynamics from different regions.

One of the salient features of the SOM is topology preservation; that is, the neighboring PEs in the feature space correspond to neighboring states in the input data. In the application of SOM modeling to the similarity index concept, the topology preserving quality of the SOM will be of added advantage, because of the fact that the neighboring PEs in the feature space will now correspond to neighboring states in the input data.

Assume X and Y are two time series generated by a system, which are embedded into two vector signals in time-using delays. Define the activation region of a PE in the SOM as the set of all input vectors (the embedded signal vectors) for which the PE is the winner based on some distance metric (Euclidean in most cases). Let *$X_{n}$* be the set of time indices of input vectors *$x_{j}$* that are in the activation region of the winner PE corresponding to the input vector *$x_{n}$* at time n. Similarly define the set $Y_{n}$.

Then the procedure to estimate the directed SOM-SI between X and Y is as follows:

1. Train an SOM using embedded vectors from both X and Y as the input.
2. At time n, find $W_{n}^{x}$, the winner PE for vector $x_{n}$, and find $W_{n}^{y}$, the winner PE for vector *$y_{n}$*.
3. To find *$R^{n}\left(X\right)$*, compute the average Euclidean distance between $W_{n}^{x}$ and all the other winner PEs in the SOM. Similarly, compute *$R^{n}\left(Y\right)$*.
4. Determine the sets *$X_{n}$* and *$Y_{n}$* for $W_{n}^{x}$ and $W_{n}^{y}$, respectively.
5. Determine the nearest PEs $W_{n,j}^{y}$ corresponding to vectors $y_{j}$, where $j\in X_{n}$. Determine the nearest PEs $W_{n,j}^{x}$ corresponding to vectors $y_{j}$, where $j\in Y_{n}$.
6. Calculate $R^{n}(X|Y)=(1/q)\Sigma_{j=1}^{q}\left\|W_{n}^{x}-W_{n,j}^{x}\right\|$, where q is the number of elements in *$X_{n}$. * Calculate $R^{n}(Y|X)=(1/q)\Sigma_{j=1}^{q}||W_{n}^{y}-W_{n,j}^{y}||$, where q is the number of elements of *$Y_{n}$*.
7. Compute the ratios,
  (2) $$\begin{array}{c} N^{n}(X|Y)=\left(R^{n}(X)-R^{n}(X|Y)\right)/R^{n}(X),\\ N^{n}(Y|X)=\left(R^{n}(Y)-R^{n}(Y|X)\right)/R^{n}(Y). \end{array}$$
8. Find *interdependencies* N(X∣Y) and N(Y∣X) as the average of $N^{n}(X|Y)$ and $N^{n}(Y|X)$ over all n.
9. Compute the SOM-SI as the difference,
  (3) χ=N(Y∣X)−N(X∣Y).

Positive values of **χ** indicate that influence of X on Y is more than the influence of Y on X, while negative values indicate the opposite. Higher magnitude of **χ** indicates a stronger coupling of the signals.

The computational savings of the SOM approach is an immediate consequence of the quantization of the input (signal) vector space. The nearest neighbor search involves *O(NM)* operations as opposed to $O\left(N^{2}\right)$ in the original SI, where M is the number of PEs. Traditionally M≪N, hence, SOM-SI offers a significantreduction in computations compared to original SI.

### 2.2. Testing the robustness of SOM-SI on multiple SOMs

To illustrate the accuracy of the SOM-based measure, we previously presented a few experimental simulations [5, 9] involving synthetically constructed linear and nonlinear interactive models. Results from each of them demonstrated the accuracy of our quantized measure, validated through statistical quantification with results from the original SI measure. For application on seizures especially, a 25×25 sized, 2-dimensional SOM grid was trained to embed all the dynamical states of an EEG attractor. SOM, being one of the most important elements of this improvised measure, one of the pre-requisites of this approach, is to ensure that: (a) for data modeling purposes, the training set captures the variance found in the dynamics of the ictal states from all the channels for a given patient and (b) the similarity indices computed using the SOM’s processing elements are independent of the SOM and the corresponding training dataset. Put in other words, pair-wise similarity indices computed on two separate SOMs should be significantly close to each other if not equal.

**Figure 1:**
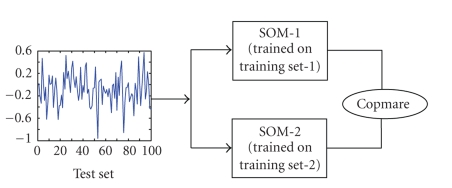
Experimental setup to compare SOM-Similarity Indices obtained from two (2) separate maps.

While the previous test results [9] were a testimony to the former, the independence of the observed interactions through similarity indices to a given SOM needed to be tested before proceeding with extensive data analysis. From the multivariate EEG data samples of an epileptic patient, two separate training sets were constructed. One of the training sets (say training set-1) consisted of portions of data sampled from the interictal, ictal, preictal, and postictal states of seizures 1 and 2. The other training set (say training set-2) consisted of data portions picked around seizure 4 and 5. Using the same normalization procedures on both the sets and with the same set of training parameters as before, two separate SOMs (called as SOM-1 and SOM-2 for convenience) were trained. Post training, the SOM-similarity indices were obtained from pair-wise analysis of interdependence among channels chosen from the ROF and LOF regions of the brain, as illustrated in Figure 1.

Test data from three (3) recording sites in right orbitofrontal region (namely, ROF1, ROF2, and ROF3) and 3 sites from left orbitofrontal regions (namely LOF1, LOF2, and LOF3) were picked from intervals surrounding seizures 4, 5, 6, 7 and seizure 11, respectively. The similarity index profiles ${\{N}^{1}(X|Y){\}}_{t}$ and ${\{N}^{2}(X|Y){\}}_{t}$ obtained from computing the SOM-SI on large intervals (say time t=1,…,T) of seizure data are quantitatively compared using the classical correlation coefficient and error-percentage as the comparison metrics. The error-percentage is computed as follows:

(4) $$\left\{e\right\}=100^{*}{\left\{\frac{N^{1}{\left(X|Y\right)}_{t}-N^{2}{\left(X|Y\right)}_{t}}{N^{1}{\left(X|Y\right)}_{t}}\right\}}_{t=1}^{T},$$

where N(X∣Y) is the normalized interdependency of *X* on *Y*. Note that the notations *X* and *Y* are used to denote the two channels of interest. Normalized error e quantifies the percentage difference between the interdependency values from SOM-2 and SOM-1, keeping interdependency value from SOM-1 as the reference. From the error population, the fraction of the absolute error values less than 20% and the fraction less than 10% are computed to determine the degree of dependence of the SOM-SI measure on the data used to train a SOM.

**Figure 2:**
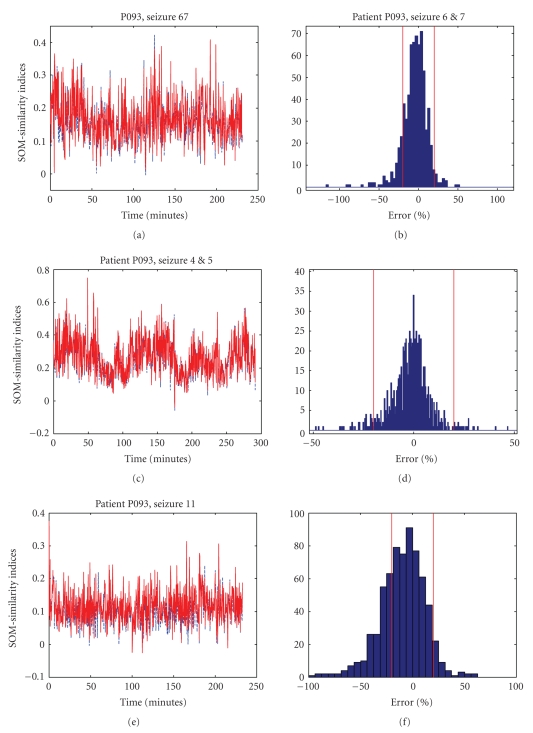
Comparing interdependencies between channels LOF3 and LOF4. Left: SOM-similarity profiles from the output of SOM-1 and SOM-2 are superimposed. Right: Histogram of the errors in %. Top: Seizure 4 and 5. Middle: Seizure 6 and 7. Bottom: Seizure 11.

For illustration, the results from analyzing the interdependency of LOF3 on LOF4 on various seizures are shown in Figure 2. The histograms correspond to the error ensembles obtained from analyzing over long seizure intervals. Qualitatively, the superimposed traces in Figure 2 indicate the extent of agreement or disagreement between the SOM-SI profiles. Table 1 compiles a summary of the agreement between the SOM-SI profiles for about 13 hours of EEG data. A large fraction of errors less than 20%, supported by a high correlation coefficient between the two SOM-SI profiles, suggests that there was very little disparity between the SOM-SI profiles from SOM-1 and SOM-2. Besides, the high percentages also seem to suggest the EEG data dynamics might not vary drastically from one seizure to another, and therefore the two SOM models produced almost identical SI results. This finding consequently supports our original belief that a well-trained SOM and a well-picked training dataset is sufficient to carry out inter-dependency analysis on all the seizures of a patient.

Overall, pair-wise analyses of the interdependency among 6 channels (15 combinations) on 5 seizures of the epileptic patient were performed on SOM-1 and SOM-2. The average correlation coefficient and the error results between the SOM-SI profiles are shown in Table 2.

Results from Table 2 indicate that in around 80% of the times, the differences between the SOM-SI results are less than 20%. This is not surprising considering that the differences are measured in percentages (3), and therefore even small discrepancies in the case of small dependency values can appear magnified. In addition, we also speculate that the discrepancies could be the outcome of the two SOMs being trained in an identical fashion instead of being fine-tuned to obtain the lowest reconstruction error in each.

In general, if the SOMs can be designed to obtain the lowest reconstruction error, by iteratively choosing the best sets of parameters, a slight improvement in the performances can be easily achieved; but as it stands, a slight discrepancy can nevertheless be always expected although it may have very little impact in the overall scheme of analysis.

## 3. SPATIO-TEMPORAL CLUSTERING MODEL

Often time series structures collected from a multi-dimensional dynamical system share similar information that reflect system wide interactions or even synchronization abilities. By definition, the word similar could mean that the information shared among a set of channels are stronger than the information they share with other channels. Such spatial similarities could possibly be transient up to a few seconds or could even stretch to several minutes or hours. As we postulated earlier, dynamial similarities in spatio-temporal behavior could be one of the driving factors to trigger certain events in biological systems. From a clinical point of view, we believe that analyzing the temporal changes in channel similarities could reveal some interesting aspects about the epileptic brain.

Similarity-based time-series clustering [10, 11] is a well-researched topic in the area of dynamical graph theory. It is an extremely useful approach to characterize spatial groupings in time sequences. Similar time sequences are typically grouped based on their mutual interactions. In this study, using the SOM-SI as a computational tool to derive the distance/similarity/proximity matrix, we propose a clustering model to dynamically analyze the spatio-temporal groupings in mutivariate time sequences.

### 3.1 Model for spatio-temporal clustering

**Figure 3:**
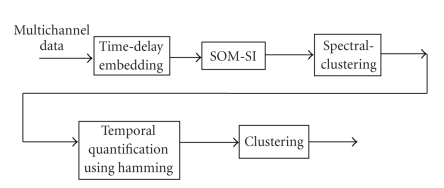
Block diagram to extract spatio-temporal groupings information in Multivariate EEG structures.

In this section, we propose a clustering approach to extract information on spatio-temporal distribution of multivariate time measurements. A 3-fold approach, consisting of spatial-discretization of the data using spectral-clustering technique [12, 13], temporal quantification using Hamming distance, followed by application of another clustering technique, is presented in Figure 3. The rational will become apparent during the explanation.

Spectral clustering is one of the many clustering methods that use subspace decomposition on data-derived affinity matrix to achieve data-clustering. Using kernel methods, the data samples are projected onto a higher dimensional space where the discriminant analysis is much easier. Projecting the data onto a feature space results in tightly formed clusters such that the between cluster entropy is maximized and the within-cluster entropy is minimized. In our study, we apply the standard spectral clustering algorithm by Ng et al. [12] to spatially cluster the similarity indices obtained by the SOM-SI technique.

Pair-wise evaluation of SOM-SI measure on all the possible combinations ($C_{2}^{N}$, where *N* is assumed to be the number of channels) of a portion of a multivariate time series leads to $k=2^{*}(C_{2}^{N})$ similarity indices in [0, 1]. *k* is multiplied by 2 because of the asymmetric nature of the SOM-SI measure. If we imagine the time series as various inter-connected nodes in a multidimensional graph, the SOM-SI similarity indices represent the affinity or rather the weights of the connection between those nodes. Therefore, we can translate them into a square matrix of size N×N, where *N* is the number of channels. Since the weighting is normalized between 0 and 1, the diagonal elements, representing the affinity of a channel with itself, are coded as 1.

However, to be able to perform spectral-decomposition on an affinity matrix, Ng’s algorithm [12] requires that the affinity matrix be square and symmetric in nature. This is because the eigen decomposition yields orthogonal column vectors (also called eigenvectors) only if the projection matrix is square-symmetric. The asymmetric matrix can be transformed to a symmetric matrix by adding it to its transpose and dividing each entry by 2. Following the eigen decomposition on the transformed affinity matrix, we have a set of labeled clusters representing the membership of the channels.

If the above procedure is repeated over consecutive time (*T*) windows (overlapping or nonoverlapping), channel groupings obtained on each time window (t=1⋯T) can be arranged in a matrix (of dimension N⁢×T) as in (5).

(5) $$\kappa_{\text{spect}}=\left[\begin{array}{cccccccccc} 3 & 2 & 2 & . & . & . & . & . & 3 & 1\\ 1 & 2 & 2 & . & . & . & . & . & 3 & 2\\ . & . & . & . & . & . & . & . & . & .\\ . & . & . & . & . & . & . & . & . & .\\ 3 & 1 & 2 & . & . & . & . & . & 1 & 2 \end{array}\right].$$

To characterize the average clustering of the channels over a longer period of time, we propose another, albeit simple, hierarchical clustering approach that uses Hamming distance to derive the proximity matrix.

### 3.2 Temporal quantification using hamming distance

We showed in the previous section that the multivariate time series can be grouped by using similarity-based clustering techniques such as spectral clustering. The spectrally clustered labels specify the groups of channels exhibiting high degree of within-cluster similarities and low degree of between-cluster similarities. Often in applications such as epileptic EEG analyses where associations last longer, it is important to identify channel groupings over a longer time-window.

State-dependent connections can be quantified by clustering rows of the $\kappa_{\text{spect}}$ matrix that are similar with each other over a longer time interval, say *T*. In this context, we propose a simple statistic that computes the relative frequency of any two channels sharing the same labels/groupings to determine the degree of similarity. In other words, in a time window of length *T*, we check the average number of times when the two channels of interest, share the same cluster label.

In an algebraic context, the above operation is equivalent to computing pair-wise Hamming distance in a time window *T*. Similarity can be quantified by subtracting the Hamming distance from 1. That is, if $d_{ij}^{\text{ham}}$ is the hamming-distance between channels “*i*” and “*j*,” similarity in probabilistic terms can be obtained as

(6) $$p_{ij}^{\text{sim}}=1-d_{ij}^{\text{ham}}.$$

Thus, computing the pair-wise similarity for all *i* and *j* combinations will result in a ***P*** matrix of size N×N (*N* is the number of channels). For convenience, we will call the matrix ***P*** the cluster-similarity matrix in all our future references.

Finally, hierarchical clustering on the cluster-similarity matrix ***P*** will yield information on the cluster groupings over a time *T*. In the context of EEG data, clustering will thus enable us to know the groups of channels that have similar behavioral structure in the brain, over a longer time frame.

## 4. EPILEPTIC EEG DATA DESCRIPTION

**Figure 4:**
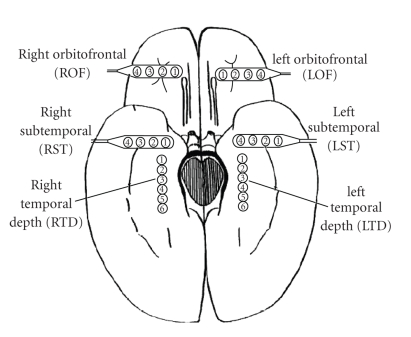
Diagram of the depth and subdural electrode montage in an epileptic brain. Electrode strips are placed over the left orbitofrontal (LOF), right orbitofrontal (ROF), left subtemporal (LST), right subtemporal cortex (RST). Depth electrodes are placed on the left temporal depth (LTD) and right temporal depth (RTD), to record hippocampus EEG activity.

Intracranial EEG signals were recorded from the hippocampus, subtemporal and frontal cortex structures of epileptic patients having a history of complex-partial and secondary generalized seizures of temporal lobe focus, using bilaterally and surgically implanted electrodes (Figure 4). The clinical motivation for the location of the electrodes was mainly to identify focal area for presurgical evaluation. Using amplifiers with an input range of ±0.6 mv, the recorded signals were converted to a narrow-band using an antialiasing filter with a cutoff range between 0.1 Hz and 70 Hz. Using an analog-to-digital converter with 10-bit quantization precision, the narrow-band signals were sampled/digitized at 200 samples/sec. Measurements involved recording EEGs from multiple sensors (28 to 32, with common reference channels) and the recordings spanned over 6 continuous days. A total of 55 seizures, of temporal lobe onset were recorded from 5 patients, in the range of 6 to 18 seizures for each patient.

The distinction of these patients from general patients with temporal lobe epilepsy is their seizures are medically refractory. In other words, these patients’ seizures cannot be controlled by the currently available anticonvulsant medications. We note that all the patients had to undergo surgery as part of their treatment.

## 5. RESULTS

In the last section, we proposed a spatio-temporal model to extract groupings from long-term multivariate recordings. In this section, we will focus on the application of that model on the epileptic intracranial EEG time series. The first part of the section will describe the details on the application of the model and the second part will discuss the results of analyses on 8 seizures, from 2 patients. With respect to selecting seizures for our analysis, the underlying reasoning was to be able to understand the following:

- complex partial types of seizures;
- how the functional relationships among different cortical sites of the brain changed over time; and
- the temporal variability of functional relationships across successive seizures.

For (c), we selected pairs of seizures that were neither too close nor too distant in time to introduce coupling from previous seizure events or external effects of many other environmental variables. Therefore, we picked pairs of seizures that were between 60 minutes and 6 hours apart only. The minimum of 60 minutes was chosen so that the second seizure was not in the postictal region of the preceding seizure. Pairs of seizures more than 6 hours apart were treated as seizures in isolation and therefore were left out of the selection.

### 5.1 Application on epileptic intracranial EEG data

The temporal changes in the spatial structure of an epileptic brain was analyzed on twenty four (24) representative channels recorded bilaterally from the orbitofrontal, temporal, and subtemporal regions on the brain. One of the fundamental requirements for analyzing the dynamics of a non linear system is to construct the state-space attractor from just a single recording of the time series. From previous studies that estimated intracranial EEG attractor size using correlation-dimension techniques [14, 15], the EEG state-space dimensionality using Taken’s embedding theorem [16] was bounded between 3 and 10. In our intracranial EEG data, the embedding dimension (*m*) and the delay (τ) were chosen to be m=10 and τ=4. The parameters were compatible with other studies [14, 15], performed on the same data. The following steps describe the procedure to track the spatio-temporal connectivity patterns in intracranial EEG data.

1. The intracranial EEG attractors were reconstructed in the high dimensional state space. On nonoverlapping 10-second epochs, one set of pair-wise interdependence values among 24 channels are computed using the SOM-SI measure.
2. The similarity indices, from every window, are translated into a symmetric similarity/affinity/proximity matrix. With the number of clusters (say $n_{1}$) specified apriori as discussed below, spectral clustering on the affinity matrix results in channels being labeled as one of the *n_1_* clusters.
3. Steps 1 and 2 are repeated for all the successive windows, representing 10-second stationary segments. However, the overall ability of the channels to associate with each other over longer time duration needs to be quantified.

On *T*: 30-minute time segments (equal to 90, 10-second windows), pair-wise Hamming-distance based cluster-similarity matrix ***P*** is computed among all the channels. The matrix elements essentially index the probability of channels to group into the same cluster over a 30-minute time interval.

Spectral clustering or any other clustering algorithm on the cluster-similarity matrix ***P*** will result in final cluster memberships. The number of clusters is fixed to *$n_{2}$* as specified below. For computing similarity indices in step 1, the epoch length of 10 seconds is chosen as a tradeoff between stationarity and sample-size requirements. Also note that the successive windows are 10 seconds apart (alternate 10-second windows) for reasons specific to computational feasibility.

We now describe step 2 in more details. The channel interdependencies obtained from SOM-SI represent the spatio-temporal correlation indices obtained by computing pair-wise similarity index among 24 channels. In spectral clustering jargon, the resulting matrix can be interpreted as an affinity matrix representing the pair-wise distances between 24 nodes. After spectral-clustering, we have a set of labeled clusters representing the membership of the channels [17]. Repeating this procedure on every 10-second window will yield a discrete-valued matrix $\kappa_{\text{spect}}$ similar to (5).

Typically, the choice for the number of clusters *$n_{1}$* in step 2 is conditioned on the significant eigenvalues. The dimensionality of the space will affect tremendously the computational complexity of the overall procedure. In our analysis, the sum of the first 3 eigenvalues typically ranged from 60% to 80% of the total variance, due to changes in seizure states. Considering this variability between epochs, and the fact that the number of clusters need to be the same for all epochs in order to be able to determine the overall grouping in channels (using cluster-similarity matrix ***P***), we fixed the number of clusters to $n_{1}=3$.

Experimental studies using nonlinear dynamics have shown [1, 2] that the quantitative descriptors of EEG exhibit seizure precursors in the form of interictal to preictal state transitions. The preictal transition time is not exactly known, however the literature [1, 2] suggests that it has a broad range of 5 minutes to 60 minutes before seizure. Therefore in step 5, as a tradeoff between state transition periods and time resolution, we choose a 30-minutes time window to characterize both the preictal and the postictal periods.

**Patient P093**

This patient had a history of complex partial seizures, localized in the mesial structures of the temporal lobe. Surgery revealed a lesion (mesial temporal sclerosis) in the right hippocampus (RTD electrodes) region. The set of 24 channels are listed below:

Channels 1 to 4: LTD3, LTD5, LTD7, LTD9,

Channels 5 to 8: RTD4, RTD6, RTD8, RTD10,

Channels 9 to 12: LST1, LST2, LST3, LST4,

Channels 13 to 16: RST1, RST2, RST3, RST4,

Channels 17 to 20: LOF1, LOF2, LOF3, LOF4,

Channels 21 to 24: ROF1, ROF2, ROF3, ROF4.

Before data analysis, a validation test was utilized to check whether application of different clustering algorithms on ***P*** would consistently result in same cluster memberships or not. For a given number of clusters *$n_{2}$*, it turned out that all the clustering algorithms including spectral clustering produced the same outputs. Therefore, we decided to choose the simple hierarchical clustering algorithm used in Matlab 6.5 owing to its graphical support.

**Figure 5:**
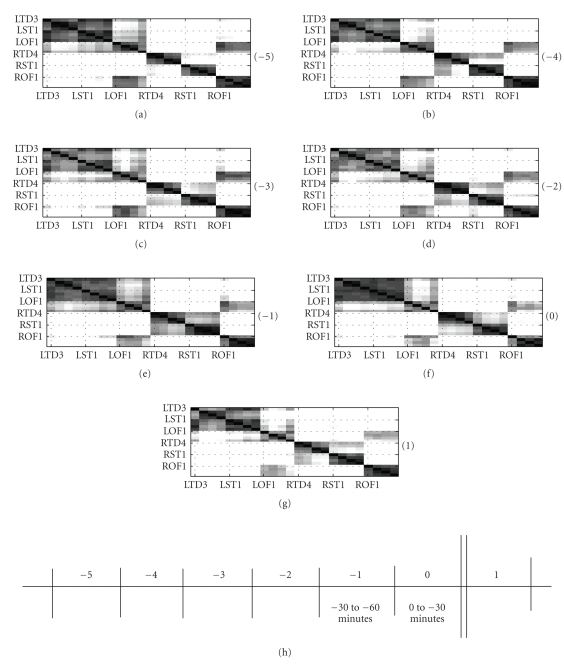
Seizure 11 of patient P093: Number in bracket indicates the 30-minute time interval when the cluster-similarity matrices were computed. The cluster-similarity matrices represent the probability that two channels share the same cluster label in a 30-minute time interval.

Cluster-similarity matrices ***P*** indicating the probability that two channels share the same grouping in a 30-minute time segment are shown gray-scale coded in Figure 5. Pre-seizure analysis on 30-minute windows is shown for up to 3 hours. Similarly, the postseizure analysis is shown for the first 30 minutes. The ability of the left side channels to have a higher tendency to group together compared to the right hemisphere channels is quite noticeable from Figure 5. In addition, the orbitofrontal lobes seem like the only brain area to have a high probability of making a cross-hemisphere grouping. On the left hemisphere, the LST and the LTD channels are consistently seen to share the same clusters.

**Figure 6:**
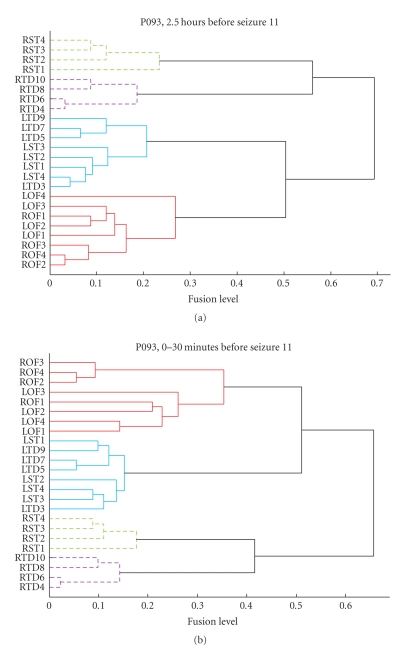
Dendrogram representation of the cluster results in Seizure 11, P093. TOP: Dendrogram corresponding to 2.5 hours before seizure. BOTTOM: Dendrogram corresponding to the 30-minute preseizure period.

To confirm the observations from Figure 5, the hierarchical clustering algorithm was applied on each of those ***P*** matrices. Figure 6 graphically illustrates two instances of the clustering outputs through dendrograms. A dendrogram is strictly defined as a binary tree with a distinguished root that has all the data items at its leaves. Conventionally, all the leaves are shown at the same level of the drawing. The ordering of the leaves is arbitrary. The heights of the internal nodes are related to the metric information (***P*** here) used to form the clustering. Using a threshold of 0.4 and the average-linkage technique to determine fusion levels, clustering was performed on a predefined number of clusters ($n_{2}$). For determining apriori the number of clusters $n_{2}$, several dendrograms were visually analyzed. There seemed to be at least 3 to 4 strong groupings among channels in most of the dendrograms. For consistency, therefore, we chose to fix the number of clusters *$n_{2}$* to 3 for all the analyses.

Both dendrograms in Figure 6 clearly translate the spatial patterns observed in the corresponding ***P*** matrices of Figure 5. The top dendrogram in Figure 6 corresponds to the 2.5-to-3 hour time window (indicated by −5) in Figure 5. It is easy to see that the dendrogram considers the RTD and the RST as isolated clusters due to their weak between-cluster fusion level. Since the number of clusters *$n_{2}$* is restricted to 3, all the remaining channels form a single large cluster. Similarly, the bottom dendrogram in Figure 6 corresponds to the P matrix indicated by −1 in Figure 5. In this case, the RST and the RTD channels group into one cluster; also well supported by a dark patch in Figure 5. This enables the LST/LTD channels and the LOF/ROF channels to group together as separate clusters.

The overall cluster configuration is listed in Table 3.

We summarize the spatial patterns at different time intervals of seizure 11 as follows.

(1) The LST and the LTD channels, in particular, exhibit a strong tendency to belong to the same group.

(2) The LOF and the ROF channels form a strong bilateral homologous connection, as seen from all the matrices in Figure 5.

(3) Relatively strong similarity can be seen between RTD and the RST channels.

(4) Common observation in all the matrices is the strong similarity between the left hemisphere channels as opposed to the right hemisphere channels. This is reflected in the ability of LOF channels to have a higher probability of sharing clusters with other left hemisphere channels, as seen in Figure 5.

(5) Interestingly, no temporal changes are seen in the spatial-patterns yet.

### 5.2. Statistical validation

The cluster configurations observed from analyzing 30-minute segments necessitates validation. Previously [9], we partially validated our model (up to the spectral clustering stage), using synthetically coupled multivariate time sequences (both nonlinear and linear). Simulations involving creation of dynamic graphs involve multidimensional time series that continuously change cluster memberships over time. Determining the average spatio-temporal groupings from a collection of multivariate time series is relatively easier to be demonstrated in linear coupling cases. However, nonlinear dynamic model constructions are extremely hard and mostly nontrivial. We therefore decided to pursue a verification of the time-averaged cluster groupings on the intracranial EEG data, using the quasisurrogate analysis technique [18–20].

Recall that the cluster groupings obtained over 30-minute time segments involve two steps. First step consists of applying spectral clustering technique on the SOM-similarity indices (computed on 10-second intracranial EEG data segments). Then similar grouping patterns among channels are extracted by using hierarchical clustering approach on the cluster-similarity matrices ***P***. In order to validate this 2-step approach, we define our hypothesis as follows.

${\text{H}}_{0}$: The average within-cluster channel interaction at each window (out of 91, 10-second windows) is not significantly different from the corresponding between-cluster channel interactions.

We propose to test this hypothesis on all the 3 ($n_{2}$) clusters separately, for every 10-second window within the 30-minute period. Within-cluster interaction is computed by averaging the pair-wise similarity indices for all the channels within a cluster. For between-cluster interaction, the pair-wise interactions among 3 channels picked randomly from each of the 3 clusters are computed. A between-cluster interaction statistic is formed by computing the average interactions from random selection of 3 channels (one from each cluster) over a number of trials. We found that this statistic follows a quasinormal distribution, implying that the within-cluster interaction value can now be compared with the mean and the variance sample estimates of the between-cluster statistic. Mathematically, we construct the z-score as follows

(7) $$Z_{t}^{i}=\frac{\left|C_{w_{t}}^{i}-\langle C_{b_{t}}\rangle \right|}{\sigma (C_{b})}\,\;t=1,2,\ldots ,90\,\;\;\text{and}\;\;i=1,2,3,$$

where $C_{w_{t}}^{t}$ is the within-cluster interaction at time “*t*”, for cluster “*i*”; $\langle C_{b_{t}}\rangle$ is the mean and $\sigma (C_{bt})$ is the standard deviation of the between-cluster interaction at time “*t*”; $Z_{t}^{i}$ reflects the z-score and is considered significant at the 95 percentile significance if $Z_{t}^{i}$> 1.96 (reject H_0_). In Table 4, the bolded value in each cell represents the number of windows (out of 91) having significant z-score in the 30-minute period corresponding to Figure 5 (P093, Seizure 11). It is easy to observe that the null-hypothesis H_0_ is rejected beyond doubt, validating the clustering results.

**Seizures 4, 5, 6 and 7:**

**Figure 8:**
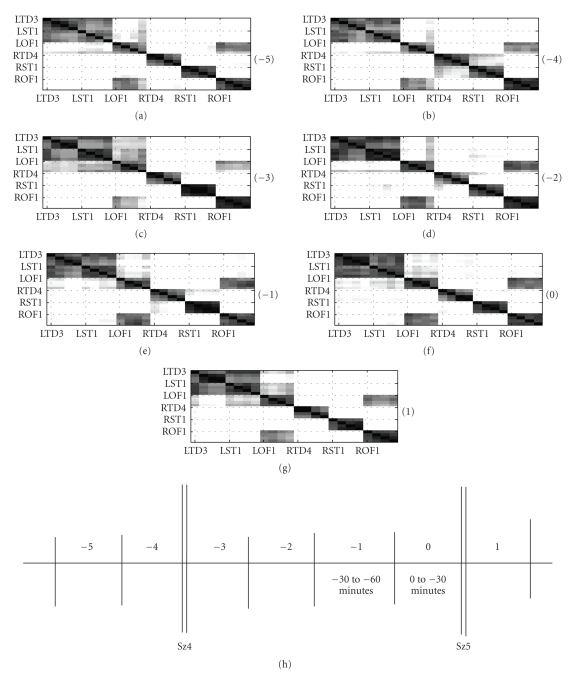
Seizures 4 and 5 of patient P093. Number in bracket indicates the 30-minute time interval when the cluster-similarity matrices were computed. The cluster-similarity matrices represent the probability that two channels share the same cluster label in a 30-minute time interval.

**Figure 9:**
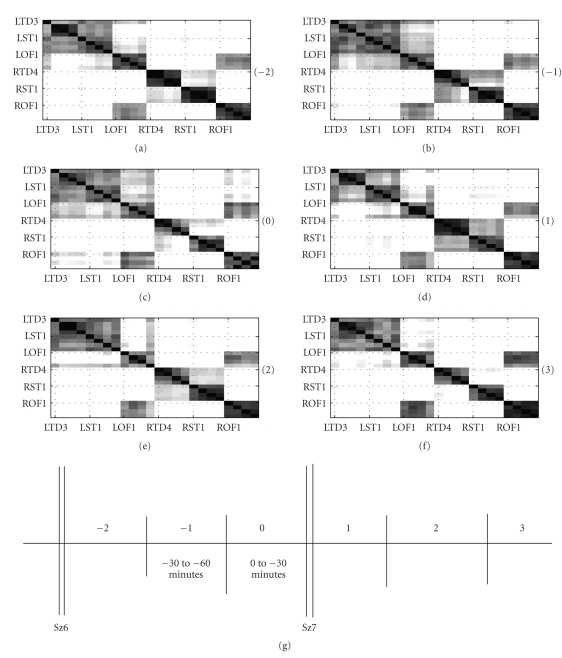
Seizures 6 and 7 of patient P093: Number in bracket indicates the 30-minute time interval when the cluster-similarity matrices were computed. The cluster-similarity matrices represent the probability that two channels share the same cluster label in a 30-minute time interval.

Spatio-temporal clustering analyses, similar to the one described on seizure 11 were performed on several other seizures, of the same patient P093. The cluster-similarity matrices ***P*** obtained from time intervals surrounding seizures 4 and 5 and 6 and 7 of patient P093 are shown in Figures 8 and 9, respectively. Channel groupings for the same are listed in tables 5 and 6, respectively. All the 4 seizures present very consistent groupings.

(1) Consistent to the observation in seizure 11, we observe the temporal depth and the subcortical regions of the left hemisphere are always grouped together.

(2) Once again, the association of ROF-LOF areas into the same cluster suggests a strong homologous connection between the orbitofrontal areas of the brain. This observation is also in agreement with those in seizure 11.

(3) The dendrograms once again presented 4 unambiguous clusters in the form of RST, RTD, LST/LTD, and LOF/ROF. The fusion levels, indicating the strength of connection between clusters, often turn out in favor of RTD and RST to be grouped separately. Owing to the fact that we have predefined the number of clusters to 3, the LST, LTD, LOF & ROF channels will consequently get grouped into one cluster.

(4) Once again, temporal changes are not very evident in the spatial patterns. However, observing Figures 8 and 9 and their corresponding dendrograms (not shown), the fusion levels and the topology of the connections change with time. These changes can only be quantified using statistical tests such as Mantel test statistics or the Double Permutation Statistics (DPS).

**Patient P092**

In this section, we present the summary results of the clustering analyses performed on patient P092 suffering from a lesion (mesial temporal sclerosis) in the medial temporal lobe structures of the right hemisphere. Channel configuration for the patient P092 is as follows:

Channels 1 to 4: LTD1, LTD3, LTD5, LTD7,

Channels 5 to 9: RTD2, RTD4, RTD6, RTD8, RTD12,

Channels 10 to 13: LST1, LST2, LST3, LST4,

Channels 14 to 17: RST1, RST2, RST3, RST4,

Channels 18 to 21: LOF1, LOF2, LOF3, LOF4,

Channels 22 to 24: ROF1, ROF2, ROF3.

Note that a separate 25×25-sized, 2-dimensional EEG-SOM grid was created to model the data dynamics of P092. Postspectral clustering analysis on 30-minute data segments led to some interesting observations.

**Figure 10:**
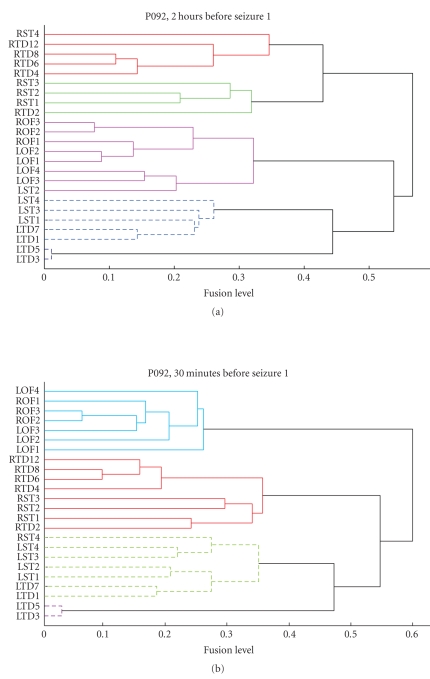
Dendrograms corresponding to P092, Seizure 1. Top: 2 hours before Seizure. Bottom: 30-minute preseizure.

Figure 10 shows the dendrograms created for seizure segments 2 hours prior to seizure 1 and 30 minutes preseizure, respectively. As before, the number of clusters ($n_{1}$) specified in the spectral-clustering step after SOM-SI block was fixed to 3. The fusion levels between most of the channel clusters is greater than 0.4, indicating a lack of strong connectivity between regions.

For the second level of clustering, as before, let the number of clusters $n_{2}$ be fixed at 3. Cluster analysis on the 30 minutes segment 2 hours prior to seizure 1 (top dendrogram in Figure 10) results in the following groups of channels:

**Cluster number 1**: LTD and LST,

**Cluster number 2**: RTD and RST,

**Cluster number 3**: LOF and ROF.

Observe the cluster formed from LTD and LST channels, in the dendrogram. It is made up of two subclusters, a large and a small cluster. The small cluster consists of only two channels, LTD (3 and 5) and fuses with the other subcluster at a very high fusion level (implying weak link). If $n_{2}$ was to be increased to 4, the clustering algorithm would classify this subcluster as an independent cluster. A detailed analysis on all seizures in P092 revealed a strong intrachannel correlation (or low fusion level) between channels LTD (3 and 5) and a weak interchannel correlation with the rest of the channels. Surrogate analysis also confirmed the imbalance by having very few rejections for the cluster consisting of LTD (3 and 5) channels. It is obvious that the average interaction (within-cluster interaction) of the largest cluster would be pulled down if there are subclusters that have a strong within-subcluster interaction, but a weak between-subcluster interaction. Consequently, the within-cluster interaction of the largest cluster can be expected to be as weak as or marginally better than the between-cluster interactions, leading to fewer rejections of the null hypothesis H_0_.

This problem can possibly be overcome by increasing the number of clusters to 4 or more. However, for consistency, we let the number of clusters *$n_{2}$* be fixed at 3 in the rest of the analyses.

**Seizures 1, 3, and 4:**

**Figure 11:**
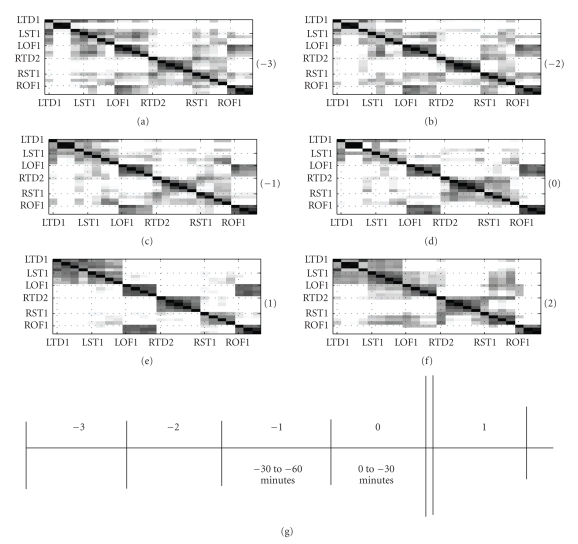
Seizure 1 of patient P092 Number in bracket indicates the 30-minute time interval when the cluster-similarity matrices were computed. The cluster-similarity matrices represent the probability that two channels share the same cluster label in a 30-minute time interval.

For illustration, the cluster-similarity matrices corresponding to seizure 1 is shown in Figure 11. Overall, the spatio-temporal clustering results for seizures 1, 3, and 4 are summarized in Tables 7 to 9.

From the cluster results of patient P092, we note the following:

(1) The nonfocal zone LTD has a strong coupling with the LST region. Correspondingly, strong affinity is observed between RTD and RST as well. These observations are consistent with the observations for P093. However, unlike in P093, we also see here that LTD connects and disconnects with several other channels, depending on the seizure state.

(2) As in P093, we observe an exclusively strong connection between ROF-LOF regions at all stages surrounding a seizure. There are few instances where the ROF breaks into a separate group. We do not have any explanation for this drift in ROF, at this point in time.

(3) Statistics from the surrogate analyses confirmed the veracity of the technique in most of the cases. As pointed out earlier, discrepancies occurred in a few instances for the clusters containing LTD (3, 5) channels.

Finally, we summarize the analysis on 2 patients and 8 complex partial seizures:

(1) Contrary to the accepted view that the seizure activity initiates in the focal zone followed by a gradual propagation to other regions, we observed that the spatial organization reflected by EEG activity exhibits either minimal or no progressive changes from the focal zone (RTD) to other zones (based on how it groups with other regions in the brain).

(2) Evidence show stronger ipsilateral connection between the LTD and LST zones compared to the connection strength between RTD-RST. Statistical analysis to check if a significant difference in intrahemisphere coupling strengths exists is needed.

(3) We also found evidence to show a strong cross-hemispheric activity by observing consistent groupings of the right and left orbitofrontal lobes at all seizure states.

(4) Patient P093 was seen to have qualitatively lesser spatio-temporal changes in its ***P*** matrices than P092 across the 30-minute analysis. It remains to be checked whether a significant change in the spatial organization before seizure is a pre-requisite to its initiation.

## 6. DISCUSSION

In this study, we applied the SOM-based similarity index measure to analyze the mutual interactions among critical areas of an epileptic brain. Based on the functional relationships, we analyzed long term structural connectivity’s related to various seizure states by proposing a spatio-temporal clustering model. On analyzing 8 complex partial seizures from 2 patients suffering from temporal lobe epilepsy, we found that the orbitofrontal regions always exhibit a strong homologous connectivity while maintaining a low relationship with other regions. The left subtemporal and the lefttemporal depth regions (nonfocal hemisphere) were identified to have a strong ipsilateral connection, regardless of seizure states. Finally, we found that the epileptic focus, namely, the right hippocampus depth region, maintained a relatively strong connection with the right subtemporal region. Interestingly, the configuration of the groupings between different regions always remained the same, regardless of whether the patient was in an interictal, preictal, or postictal state although the inter-region connectivity strengths seemed to vary slightly across states.

So far, because of the data size, we were constrained to analyze only on 8 seizures from 2 patients. Future effort in this direction would be to apply the proposed approach on a larger set of seizures and more patients. In addition, since we analyzed only complex partial seizures, it would be worthwhile to check the cluster grouping in other types of seizures such as partial secondary generalized and sub-clinical seizures.

Recall from the results that certain channels were always grouped together regardless of the seizure states. This raises a question if this pattern is unique to an epileptic patient, and therefore be considered as a blueprint of seizures. While it is almost impossible to obtain intracranial EEG on normal subjects, one plausible way to answer this speculation would be to apply the proposed clustering approach on scalp EEG data from normal subjects and then analyze the differences in groupings with that of scalp EEG obtained from seizure patients.

One of our other main objectives in this study was to develop engineering tools to determine spatio-temporal groupings in a multivariate epileptic brain. We proposed a similarity-based clustering approach and used it to extract hidden structures from an epileptic brain. One of the obvious limitations with any clustering approach is determining the optimal number of clusters. Techniques to address cluster size have been researched, without much success. In eigenvector-based methods such as spectral clustering, cluster size can possibly be approximated to be equal to the number of eigenvectors corresponding to significant eigenvalues. In multiple datasets however, the optimal cluster size need not have to be the same across different datasets rendering cluster comparisons weak. In our approach, we analyzed a large number of data sets and empirically, fixed the cluster size to 3. This may not be an efficient or a systematic approach to tackle the problem. Theoretic efforts are needed to develop a mathematical criterion that allows us to determine a fixed cluster size, suitable to all groups of data. Besides, exploring tools better than clustering to unravel hidden patterns in multidimensional time sequences would be very beneficial.

## Figures and Tables

**Figure 7:**
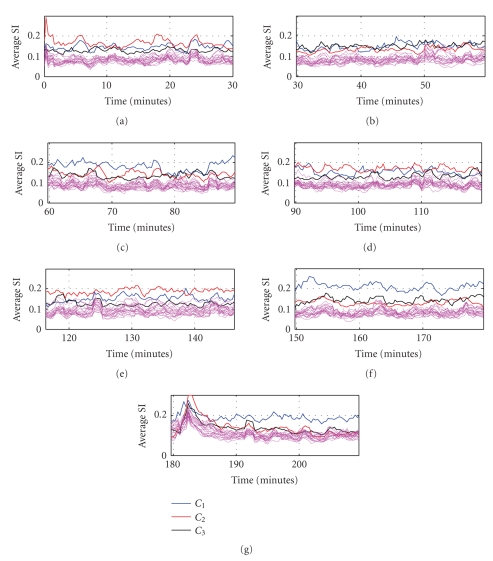
Statistical validation of the clustering results. In each panel, thick lines are used to represent the profiles of the three clusters in a 30-minute time interval. The thin lines are the surrogate profiles indicating between-cluster interactions. Cluster veracity can be visually verified by observing that amplitudes representing within-cluster interaction for cluster profiles are mostly higher that the amplitudes representing between-cluster interaction for surrogate profiles, at each time instance.

**Table 1:** Quantitative comparisons between the SOM-SI profiles obtained from SOM-1 and SOM-2. LOF3 and LOF4 data was projected on each of the SOMs and then the SOM-SI measure was applied to analyze the dependency of LOF3 on LOF4.

Interdependency N(LOF3∣LOF4)	Correlation Coefficient (%)	Fraction of error less than 20%	Fraction of error less than 10%
Seizure 6 and 7	95.74	0.8504	0.5597
Seizure 4 and 5	98.45	0.9234	0.7543
Seizure 11	91.59	0.6452	0.3614

**Table 2:** Summary of the comparisons between the SOM-SI profiles from SOM-1 and SOM-2. Each row represents the statistics (mean and variance) of pair-wise SOM-SI analyses of the epileptic EEG data from 6 channels (15 combinations).

	Correlation Coefficient (%)	Fraction of error less than 20%	Fraction of error less than 10%
Seizure 6 and 7	94.32±2.85	0.79±0.1	0.54±0.12
Seizure 4 and 5	97.46±1.08	0.91±0.06	0.73±0.12
Seizure 11	93.24±2.06	0.71±0.08	0.41±0.07

**Table 3:** Spatio-temporal groupings as obtained for seizure 11 of patient P093.

P093, Seizure 11	C_1_	C_2_	C_3_
Preseizure, (2.5–3 hrs)	RTD	RST	LTD, LST, LOF, ROF
Preseizure, (2–2.5 hrs)	RTD, RST	LOF, ROF	LTD, LST
Preseizure, (1.5–2 hrs)	RTD, RST	LOF, ROF	LTD, LST
Preseizure, (1–1.5 hrs)	RTD, RST	LOF, ROF	LTD, LST
Preseizure, (30 mins–1 hr)	RTD, RST	LOF, ROF	LTD, LST
Preseizure, (0–30 mins)	RTD, RST	LOF, ROF	LTD, LST
Postseizure, (30 mins–1hr)	RTD, RST	LOF, ROF	LTD, LST

**Table 4:** P093, Seizure 11: Over each 30-minute (91 samples total) window, number of times the within-cluster interaction is greater than between-cluster interaction, at 95% significance level.

P093, Sz 11	−5	−4	−3	−2	−1	0 (Sz)	1
C1	1	1	0.91	0.95	0.99	1	0.93
C2	0.82	0.89	0.96	0.91	0.89	0.85	0.98
C3	0.95	0.55	0.80	0.70	0.46	0.46	0.97

**Table 5:** Spatio-temporal groupings as obtained for seizures 4 and 5 of patient P093.

P093, Seizure 4 and 5	C_1_	C_2_	C_3_
Preseizure 4, (30–60 mins)	RTD, RST	LOF, ROF	LTD, LST
Preseizure 4, (0–30 mins)	RTD, RST	LOF, ROF	LTD, LST
Postseizure 4, (0–30 mins)	RTD	LTD, LST, LOF, ROF	RST
Postseizure 4, (30 mins–1 hr)	RTD	LOF, ROF	LTD, LST, RST
Preseizure 5, (30 mins–1 hr)	RTD	LTD, LST, LOF, ROF	RST
Preseizure 5, (0–30 mins)	RTD	LTD, LST, LOF, ROF	RST
Postseizure 5, (30–1 hr)	RTD	LTD, LST, LOF, ROF	RST

**Table 6:** Spatio-temporal groupings as obtained for seizure 6 and 7 of patient P093.

P093, Seizure 6 and 7	C_1_	C_2_	C_3_
Postseizure 6, (0–30 mins)	RTD, RST	LTD, LST	LOF, ROF
Preseizure 7, (30 mins–1 hr)	RTD, RST	LTD, LST	LOF, ROF
Preseizure 7, (0–30 mins)	RTD	LTD, LST, LOF, ROF	RST
Postseizure 7, (0–30 mins)	RTD	LTD, LST, RST	LOF, ROF
Postseizure 7, (30 mins–1 hr)	RTD	LTD, LST, LOF, ROF	RST
Postseizure 7, (1 hr–1.5 hrs)	RTD	LTD, LST, LOF, ROF	RST

**Table 7:** Spatio-temporal groupings as obtained for seizure 1 of Patient P092.

P092, Seizure 1	C_1_	C_2_	C_3_
Preseizure, (1.5–2 hrs)	RTD, RST	LTD, LST (1, 3, 4)	LOF, ROF, LST (2)
Preseizure, (1–1.5 hrs)	RTD	LST, RST, LOF, ROF, LTD (1, 7)	LTD (3, 5)
Preseizure, (30 mins–1 hr)	RTD, RST	LTD, LST	LOF, ROF
Preseizure, (0–30 mins)	RTD, RST	LTD, LST	LOF, ROF
Postseizure, (0–30 mins)	RTD, RST	LTD, LST	LOF, ROF
Postseizure, (30–1 hr)	RTD	LTD, LST, LOF, RST	ROF

**Table 8:** Spatio-temporal groupings as obtained for seizure 3 of Patient P092.

P092, Seizure 3	C_1_	C_2_	C_3_
Preseizure, (1.5–2 hrs)	RTD	LST, LTD, RST	LOF, ROF
Preseizure, (1–1.5 hrs)	RTD	LST, LTD, RST	LOF, ROF
Preseizure, (30 mins–1 hr)	RTD, RST	LST, LTD	LOF, ROF
Preseizure, (0–30 mins)	RTD, RST	LST, LTD	LOF, ROF
Postseizure, (0–30 mins)	RTD, RST	LST, LTD	LOF, ROF
Postseizure, (30–1 hr)	RTD, RST	LST, LTD	LOF, ROF

**Table 9:** Spatio-temporal groupings as obtained for seizure 4 of Patient P092.

P092, Seizure 4	C_1_	C_2_	C_3_
Preseizure, (1.5–2 hrs)	RTD, RST	LST, LTD	LOF, ROF
Preseizure, (1–1.5 hrs)	RTD, RST	LST, LTD	LOF, ROF
Preseizure, (30 mins–1 hr)	RTD	LST, LTD, RST	LOF, ROF
Preseizure, (0–30 mins)	RTD, RST	LST, LTD	LOF, ROF
Postseizure, (0–30 mins)	RTD, RST	LST, LTD	LOF, ROF
Postseizure, (30–1 hr)	RTD, RST	LST, LTD	LOF, ROF
